# Efficacy of Delta Plate in Condylar Fracture: a Case Series with Review

**DOI:** 10.30476/DENTJODS.2022.94746.1806

**Published:** 2023-03

**Authors:** Aniket Sarkar, Lahari Banerjee, Samiran Ghosh

**Affiliations:** 1 Dept. of Oral and Maxillofacial Surgery, Haldia Institute of Dental Sciences and Research, Haldia, West Bengal, India; 2 Consultant Oral and Maxillofacial Surgeon, Kolkata, India; 3 Dept. of Oral and Maxillofacial Surgery, Guru Nanak Institute of Dental Sciences and Research, Panihati, Kolkata, India

**Keywords:** 3D plate, Mandibular Condyle, Osteosynthesis of condylar fracture

## Abstract

Open reduction and internal fixation (ORIF) of mandibular condylar fracture with a three dimensional stabilization has been a controversial topic in oral and maxillofacial surgery. Miniplates and many 3D plates have been used till now for fixation of condylar fracture and delta plate is one of them. Present literature has less evidence about which one is superior over another. We have tried to evaluate the clinical performance of the delta miniplate in this study. A total of 10 patients presenting mandibular condylar fracture were treated by ORIF using delta miniplate. Dimensional details were measured of 10 dry human mandibles. At the end of 1-year follow-up period, all patients had satisfactory results, both clinically and radiologically. Delta plate showed better stability in the condylar region and less complication associated with plating system.

## Introduction

A mandibular condyle fracture comprises 25 to 45% of all mandibular fractures [ 1 - 2 ]. Direct and indirect forces in the condylar region most commonly cause unilateral or bilateral subcondylar fracture [ 3 ]. Anatomically, the mandible dissipates the forces to the condylar neck, which is more susceptible to fracture, and therefore prevents forces from being transferred to the cranium. This increases the frequency of mandibular condylar fracture [ 4 ].

The narrow bone anatomy in the condylar neck does not often provide the necessary surfaces for positioning of multiple hardware according to the stress lines. The stresses in the thin condylar neck area increase significantly in comparison to the broader condylar base. Stable three-dimensional fixation is very important as this may lead to non-union, fibrous union, or temporomandibular disorders from interfragmentary mobility [ 5 - 6 ].

3D Delta plates have been designed to adapt to and neutralize strain during ORIF of condylar fractures and have the advantage of being smaller in size. The aim of this study was to address the efficiency of the delta plate for the fixation of the condylar fracture.

## Case Presentation

**Figure 1:**
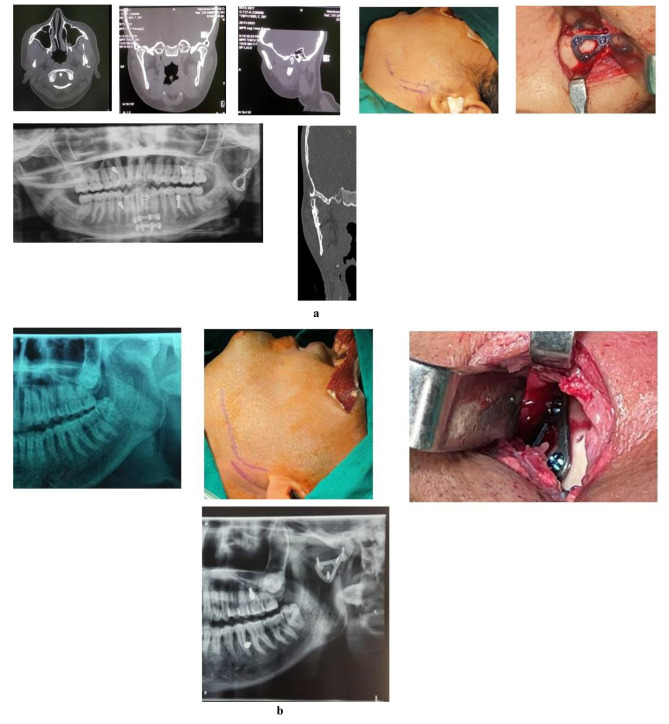
**a:** Preoperative ct scan in axial, coronal and sagittal view. Retromandibular incision marked open reduction and internal fixation (ORIF) done with delta plate postoperative orthopantomogram, **b:** Preoperative orthopantomogram retromandibular incision marked. Open reduction and internal fixation (ORIF)done with delta plate postoperative orthopantomogram

**Figure 2:**
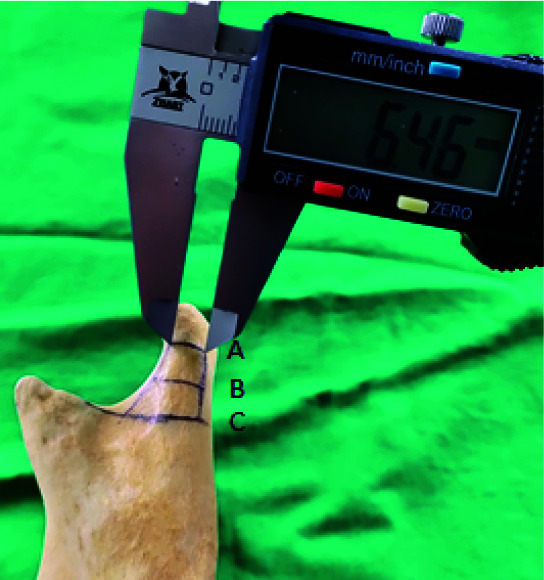
Dimensions of condylar neck region measurement done with vernier calipers in the dry mandible

**Figure 3:**
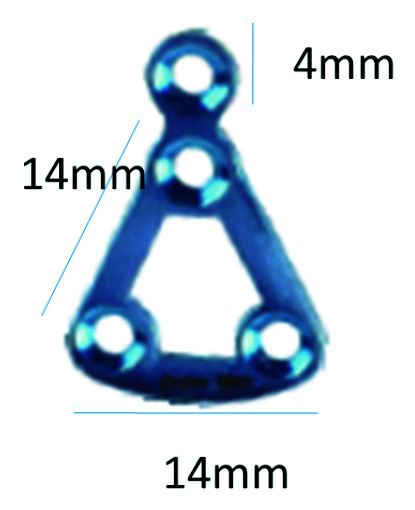
Dimensions of indigenous titanium delta plate

**Figure 4:**
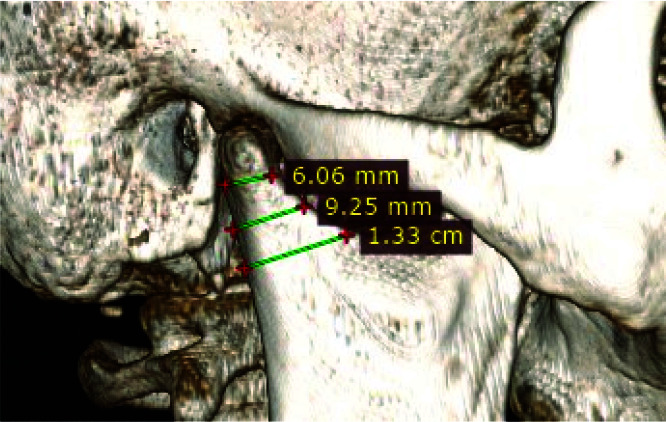
Dimensions of condylar neck region in computed tomography scan

In this retrospective review, we did a case series of condylar fractures on 10 patients where all patients had clinical symptoms including deranged occlusion, reduced interincisal distance, and restricted mandibular movements. All the patients were treated with ORIF using a delta plate. In all cases, preoperative and postoperative clinical and radiological examinations were done (Figure 1a and b). We diagnosed the condylar fractures with the help of orthopantomogram and computed tomography scan preoperatively. We have also measured the dimensional details of the anteroposterior width of the condylar neck, on 10 dry human mandibles and also the dimensional details of the plate (Figures 2 and 3). The measurement details were used for preoperative assessment in treatment planning. Simulation of these measurement details was done with the computed tomography scan with the help of Radi-Ant DICOM Viewer software (v2021.1) (Figure 4).

**Figure 5:**
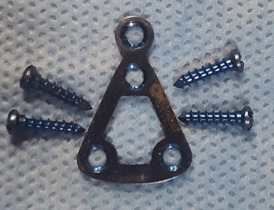
Indigenous titanium delta plate with four screws

**Figure 6:**
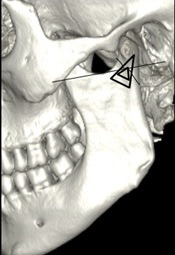
Easy hypothesis for delta plate fixation

The mandibular condyle was approached by a retromandibular incision in all patients. An ORIF of a condylar fracture using the triangular-shaped delta plate provides stability in three dimensions. In the delta plate, the lines of tensile and compressive stress distribution run parallel to both sides of the plate and the base is oriented toward the angle of the mandible. The plate consists of two longitudinally arranged holes at the top, and two more holes are located at the two corners of the base of the plate (Figures 5 and 6).

The surgical approach for the patients in the open reduction group was retromandibular. In all cases, delta plate was used for fixation at different levels of the condylar neck fracture. No postoperative occlusal derangement was noted. All patients had optimal jaw movement without any deviation. Postoperative radiographs showed satisfactory healing in all patients. After a 1-year follow-up period, there were no signs of screws loosening or secondary displacement of fractured segments.

In all the 10 cases, it showed acceptable results in relation to ramal height maintenance and proper angular reduction; though we feel a larger sample size would have provided a more reliable outcome to make a comment on (Table 1).

**Table 1:** Shows the average of the measurement in the dry mandible

Landmark	Distance(mm)
A	6-7
B	8-9
C	13-15

## Discussion

According to Anirudhan *et al*. [ 7 ] and Lauer *et al*. [ 8 ], compressive and tensile stress lines run through the bone along both side of the delta plate. It has a cross-section of 1×2.5mm and finite element analysis shows the distribution of tensile strain along the anterior border of the delta plate. 

The ease of positioning of delta miniplate and securing two screws through the vertically placed holes in the narrow region of the proximal segment is one of the major benefits of using it [ 9 ]. A delta plate does not interfere in the fracture healing process and practically, it is almost impossible for the plate to break or fail as a result of the loosening of the retaining screws [ 9 ]. With this design of the delta miniplate, it is possible to relieve the fracture segment of the condyle from any postoperative stress better than other mini plates, which allows for a faster healing and reduced complications after surgery [ 8 , 10 - 12 ].

Ganguly *et al*. [ 13 ] stated that compared to conventional mini plates, delta plates provide better results in terms of operating time, mouth opening, and biting efficiency. The geometry and the design of the plate provide better stability and it allows the implant to easily accommodate the anatomy of the fracture, resulting in minimal risks and complications intraoperatively. A week of immobilization was sufficient to allow for early rehabilitation and functional loading.

The study by Arjun Ahuja *et al*. [ 14 ] concluded that delta plates are superior from the perspective of handling, ease of adjustment, and takes less time for adaptation. It is also a cost-effective option from the patient’s perspective. The delta miniplate has the highest values (17.3 N/m) in terms of rigidity. In terms of posterior-anterior direction, the delta plate is considered as best. A specific geometry of the delta plate gives three-dimensional stability and optimal leverage [ 15 ]. 

In terms of hardware volume, delta miniplate has a noticeably smaller volume than other mini plates [ 10 ]. Strain in bone is remarkably high around the screws in the mesial fragment of the delta miniplate when compared to other mini plates [ 10 ]. The improvement in the protrusive movement of the mandible was found to be gradual and better in the delta miniplate than in other miniplates [ 16 ]. Evaluation of primary stability in the region of the fracture line is determined by the opportunity to take the stress from bone to plate and distribute it to numerous screws. Better distribution of tension throughout the plate and horizontal arrangement of screws in the distal segment provide better secondary stability in the area of fixation.

In our experience, the delta miniplate is easy to adapt in the condylar neck region with less operating time, more internal stability, less stress related to screws, and has an acceptable outcome. It can withstand muscular forces with stress concentration in the condylar neck region. Patient consent form was signed and taken from each of the patients.

## Conclusion

Delta plate provides better stability due to its geometry and configuration at the condylar neck region. There are several advantages associated with this small profile osteosynthesis plate, including its ability to neutralize the changing strains at the lateral, anterior, and posterior borders and the fact that the base acts as a compression plate. This plating system is associated with very few complications but it also has some disadvantages such as the length of the delta plate and the adaptability with the curvature in the condylar neck region.

## Acknowledgment

Written informed consent was obtained from the patients for publication of this research article and accompanying images.

## Conflict of Interest

The authors declare that they have no conflict of interest.
